# Editorial: NETosis 2: The Excitement Continues

**DOI:** 10.3389/fimmu.2017.01318

**Published:** 2017-10-20

**Authors:** Luis E. Muñoz, Mariana J. Kaplan, Marko Radic, Martin Herrmann

**Affiliations:** ^1^Department of Internal Medicine 3 – Rheumatology and Immunology, Friedrich-Alexander-University Erlangen-Nürnberg (FAU), Universitätsklinikum Erlangen, Erlangen, Germany; ^2^Systemic Autoimmunity Branch, National Institute of Arthritis and Musculoskeletal and Skin Diseases, National Institutes of Health, Bethesda, MD, United States; ^3^Department of Microbiology, Immunology and Biochemistry, University of Tennessee Health Science Center, Memphis, TN, United States

**Keywords:** neutrophil extracellular traps, inflammation, resolution of inflammation, chromatin, obtruction

**Editorial on the Research Topic**

**NETosis 2: The Excitement Continues**

## Introduction

Chromatin externalization to form extracellular traps has first been described in neutrophils (1) but can also be observed in mast cells (Mollerherm et al.) as well as in other myeloid cells like eosinophils and basophils (2). DNA externalization-based defense emerged more than 1 billion years ago. Sentinel cells of the social ameba *Dictyostelium discoideum* release redundant mitochondrial DNA and in an altruistic manner chromatin that sequester colony invading bacteria (Zhang and Soldati). Leukotoxic hypercitrullination and deficient mitophagy can initiate mitochondrial DNA expulsion (Konig and Andrade). These processes can be discriminated from canonical NET formation (1). However, since they cause DNA externalization they could be considered non-canonical forms of NET formation. Although the generation of reactive oxygen species is reportedly involved in several pathways of NET formation it is not strictly required for others (3). Furthermore, under hypoxic conditions, the PMA-induced NET formation is reported to be reduced, though not abrogated (Branitzki-Heinemann et al.). This editorial summarizes the collection of articles of the research topic “NETosis 2, the excitement continues.”

## To Die or Not to Die

There is ongoing discussion whether the neutrophils are still viable when they release their chromatin and if they release chromatin at all. There are reports that claim the cells mainly externalize mitochondrial DNA (Yousefi and Simon). Some investigators question the notion that NETs form by cytolysis. They argue that it seems inconsistent with neutrophil “containment” processes (Malachowa et al.). Furthermore, the term “NETosis” has been criticized by some researchers who prefer the term “NET formation.”

Surveying the published data, including those published in this special issue, we want to summarize the current knowledge (I) Depending on the stimulus, NET formation can be a lytic death process eventually leading to disintegration of the neutrophil or a process where neutrophils remain migratory and impermeable to ionic dyes; (II) these processes have been observed *in vitro* as well as *in vivo*; (III) the inducer and its concentration determine, at least in part, these processes as well as the content of the NETs. For purposes of this review, we will use the term NET formation rather than NETosis to include the various forms of this cellular processes as described by various research groups.

## Inducers of NET Formation

NET formation is favored by the higher pH at the borders of inflamed areas (Maueroder et al.). The neutrophil granule-derived enzyme peptidylarginine deiminase type 4 has been proposed to be involved in the formation of NETs and its pharmacological inhibition suppresses chromatin decondensation and NET formation following certain types of stimuli (Kusunoki et al.). In response to hyphae of *Candida albicans* or immobilized β-glucan neutrophils that are associated with the extracellular matrix rapidly form NETs independent of their ability to perform an oxidative burst (O’Brien and Reichner). The pathway of immune complexes to induce NET formation involves cross-linking of the receptor for IgG Fc fragments FcγRIIIb, the TGF-β-activated kinase 1, and activation of MEK/ERK signaling (Aleman et al.). NET formation is also triggered by interaction of neutrophils with platelets (Carestia et al.) and by a complex interplay between neutrophils and bacterial lipopolysaccharides (LPS). Both “suicidal” and “vital” NET formation depend on the bacterial origin of the LPS and are modulated by the presence or absence of platelets (Pieterse et al.). NETs generated in the presence of propylthiouracil display a disordered phenotype which renders them resistant to treatment with DNaseI, and have been proposed to favor the generation of ANCAs (Soderberg and Segelmark). During gestation, the capacity to form NETs increases and involves chorionic gonadotropin, G-CSF, and estrogen (Giaglis et al.).

## Inhibitors of NET Formation

The increased capacity to form NETs during gestation is controlled by the inhibitory activity of progesterone. This hormone reduces the nuclear translocation of the neutrophil elastase leading to decreased NET formation (Giaglis et al.). Furthermore, Raloxifene, a selective modulator of the receptor for estrogen prevents NET formation induced by PMA (Flores et al.).

## NET Components

The NET backbones mainly consist of chromatin with modified, often citrullinated, histones and is overall similar in most NETs reported. However, there are considerable differences in their protein cargo (Mitsios et al.). These proteins may either be derived from the neutrophils’ granules, from the vicinity of the NETs or from bystander cells. Neutrophils are endowed with a “tool kit” for alternative complement activation. They secrete properdin and deposit C3-derived complement fragments on NETs and on NET-bound bacteria (Yuen et al.). Since NETs are endowed with a potent toxic and proteolytic armament they need regulatory components to limit tissue injury. Hence, the secretory leukocyte protease inhibitor SLPI is bound to NETs. However, SLPI-NETs carry a certain risk; interacting with plasmacytoid dendritic cells, they induce type 1 interferon *in vitro*, cytokines that have been associated with the development of various autoimmune conditions (Majewski et al.). Extracellular histones are cytotoxic to endothelial cells and thus may contribute to septic pathology and death. The NET component long pentraxin PTX3 protects against histone-mediated cytotoxicity (Daigo et al.).

## The Roles of Histones

Histones are endowed with bactericidal and fungicidal activities. This comes at the prize of bystander cytotoxicity toward endothelial cells. Thus, extracellular histones are partially responsible for tissue damage and septic death. To dampen overwhelming histone toxicity, NETs can be decorated with the long pentraxin PTX3 that limits histone-mediated tissue damage (Daigo et al.). Antibodies against posttranslational modified, citrullinated histones are pathognomonic for patients with rheumatoid arthritis (Neeli and Radic). In contrast to humans, some autoimmune mice are tolerant to citrullinated histones (Dwivedi et al.), a finding that highlights differences in PAD4 expression in human versus mouse antigen-presenting cells.

## Antibodies Binding NETs and Their Components

Deimination is a physiological process that is amplified at sites of inflammation. However, only individuals with genetic predispositions for rheumatoid arthritis develop antibodies against deiminated proteins (ACPAs) (Corsiero et al.). Patients with malaria show circulating NET-binding P-ANCA (anti-MPO) and to a lesser extend C-ANCA (anti-PR3) which does not react with NETs. This supports the notion that NETs are involved in the etiopathogenesis of this, often fatal, disease (Boeltz et al.). Excessive NET formation in Balb/c wild type mice leads to generation of MPO-ANCA production *in vivo* (Kusunoki et al.). Importantly, the autoantibody binding to NETs may not only contribute to immune pathology but may aid in the clearance of NETs (Soderberg and Segelmark).

## Methods for the Analysis of NETs

In general, NETs can be detected by staining of DNA and its co-localization with granular and modified nuclear proteins in web-like, spiky or cloudy structures, exceeding the size of a neutrophil. In neutrophil-rich areas like inflamed synovium or densely infiltrated tissues, these NETs tend to aggregate (4, 5) and form extended chromatin clumps decorated with an effective pathogenocidal armament (Brinkmann et al.). When exploring intravascular NET formation in disease, the triad neutrophils, platelets, and endothelium should be analyzed (Kazzaz et al.). For *in vitro* and *ex vivo* assays, the medium composition has to be precisely reported and strictly controlled during the entire assay and not just at its beginning (Maueroder et al.). Detection of deiminated histones is often used as an indication of NETosis but it should be stressed that the current methods for detection of citrullinated proteins require further optimization (Daigo et al.).

## Barrier Function of Aggregated NETs

NETs generated at high neutrophil densities often aggregate and form extended matted structures (4, 5) decorated with a plethora of bactericidal and fungicidal molecules. Though these structures are based on a DNA meshwork, they only display a limited sensitivity against DNA degrading enzymes. Indeed, gouty tophi, an aggregate of NETs and MSU crystals, can persist in human tissues for several years where DNA is still detectable. In the periphery of necrotic areas, necrotic cells may recruit neutrophils and initiate and/or facilitate the formation of NET-based surrogate barriers [Bilyy et al.; Biermann et al.].

## Intravascular NET Formation and Vascular Diseases

Intravascular NETs have pro-coagulant activities associated to several pathologies (Kimball et al.). As in SLE, in ANCA associated vasculitis, the degradation pathways of NETs are perturbed. Due to low DNaseI activity, the degradation of intravascular NETs is slow leading to an increased amount of circulating NETs (Soderberg and Segelmark). This phenomenon may also contribute to the pathogenesis of severe malaria (Boeltz et al.). Similarly, NETs are discussed to contribute to the thrombotic pathologies observed in patients with cancer (Olsson and Cedervall).

After bone marrow transplantation, the capacity to form NETs is reportedly reduced for up to 200 days (Glenn et al.). In an animal model of paw edema by injection of nanodiamonds into wild-type mice and in those with deficient capacity for oxidative burst and NET formation (Ncf1** mice), the inflammatory response resolves in the former and becomes chronic in the latter as a result of the failure to dampen the neutrophil-driven inflammation (Biermann et al.). Importantly, ANCA in general are reported to drive immune complex-mediated pathologies but they may also aid in the clearance of circulating NETs or NET remnants (Soderberg and Segelmark).

## NET Formation and Extravascular Disease

Though NETs have initially been described as mechanism of bacterial defense, they must be considered as double-edged swords of innate immunity (Yang et al.). NETs are involved in a plethora of pathological conditions including autoimmunity, atherosclerosis, cancer, etc. (Mitsios et al.). Excess neutrophil recruitment to the alveolar space and NET formation in lungs reportedly cause inflammation and asthma (Akk et al.). NETs can easily expand in the pulmonary alveoli and cause lung injury. DNA disintegration by DNase, neutralization of NET proteins, anti-histone antibodies, and protease inhibition may alleviate NET-associated pathologies (Porto and Stein). Raloxifene, a selective modulator of the estrogen receptor inhibits the NET-based killing of the leading human bacterial pathogen MRSA (Flores et al.). Furthermore, it was recognized that viruses induce NETs and target these for immune evasion (Schonrich and Raftery). Harming of the blood–brain barrier and of neural cells by NETs was observed in humans with Alzheimer’s disease and in a murine model of this disabling disease (Pietronigro et al.).

## Therapeutic Interventions

NETs are discussed as a source of the citrullinated autoantigens pathognomonic for patients with rheumatoid arthritis and for the DNA observed in tissues and in the circulation of patients with SLE. Consequently, PAD4 is proposed to be a potential therapeutic target for these chronic inflammatory rheumatic diseases (Konig and Andrade). The synthetic peptide P140/Lupuzor™ selectively modulates chaperone-mediated autophagy but not NET formation in sensu stricto (Ramirez et al.). As high fat diet reportedly increases the formation of NETs dietary intervention and reduction of fat intake may be beneficial in NET-associated disorders (Moorthy et al.).

Targeting NET formation as well as NET-associated chromatin decondensation may delay the pathogenesis of Alzheimer’s disease (Pietronigro et al.), the early inflammatory responses to Sendai virus infection (Akk et al.), and the disseminated intravascular coagulation observed in severe forms of malaria (Boeltz et al.). As already established for patients with cystic fibrosis, the clearance with recombinant human DNaseI of the NET-associated DNA, the neutralization of NET-borne proteins using anti-histone antibodies, as well as inhibitors for NET-bound proteases are discussed as therapeutic options for pulmonary diseases involving alveolar NETs in Porto and Stein.

From the papers included in this Research Topic, it is evident that the field of NET research is now more mature and sophisticated than even just a few years ago. Physiological and disease conditions that induce abundant NET formation are now firmly established and experimental methods for detection of NETs *in vivo* and *in vitro* have been carefully defined. It is the hope of the authors that the combined efforts presented here will contribute to further shape the consensus in the field, energize efforts to understand NET biology, and lead to novel therapies for major human disorders that present with abnormal NET release or impairments in NET degradation.

## Author Contributions

All authors wrote and revised the manuscript.

## Conflict of Interest Statement

The authors declare that the research was conducted in the absence of any commercial or financial relationships that could be construed as a potential conflict of interest.
